# Charles Bonnet Syndrome Related to a Pituitary Adenoma: A Case Study in a Tunisian Woman

**DOI:** 10.1155/2023/9979128

**Published:** 2023-04-03

**Authors:** Haifa Ghabi, Amira Maamri, Ahlem Hajri, Haifa Zalila

**Affiliations:** Razi Hospital, Tunis, Tunisia

## Abstract

According to the International Statistical Classification of Diseases and Related Health Problems 11th Revision (ICD-11) definition, “Charles Bonnet Syndrome” (CBS) refers to the experience of complex visual hallucinations in a person who has experienced a partial or complete loss of vision. Pituitary adenoma is a rare situation that may be associated with CBS. We report a case of CBS in a Tunisian 44-year-old female with pituitary macroadenoma. The patient reported visual hallucinations which persisted after transsphenoidal adenomectomy. She had no prior psychiatric history. She did not take any medication that could produce hallucinations. After Olanzapine initiation, hallucinations were completely resolved. It is the first Tunisian case of CBS reported in English language. This peculiar condition seems to be under-recognized in our country. Clinicians should be aware that visual hallucinations may concern patients without psychiatric disorders.

## 1. Introduction

Charles Bonnet Syndrome (CBS) is defined by the association of four conditions: (1) persistent or repetitive complex visual hallucinations, (2) insight into the hallucinations, (3) no other types of hallucinations, and (4) no primary or secondary delusions [1]. CBS associated to pituitary macroadenoma is rarely reported in the literature [2]. We report a case of CBS in a Tunisian 44-year-old female with pituitary macroadenoma. The patient reported visual hallucinations which persisted after transsphenoidal adenomectomy.

## 2. Case Presentation

A 44-year-old Caucasian female was referred to our department of psychiatry by her neurosurgeon, after having been operated on a pituitary macroadenoma compressing the optic chiasm, for visual hallucinations.

She was married. She had no prior psychiatric history. She did not take any medication that could produce hallucinations.

On March 2019, the patient underwent transsphenoidal adenomectomy.

In the first postoperative day, the patient reported the persistence of visual hallucinations and was referred to our department of psychiatry one week later.

Upon arrival to our department, the patient was cooperative, did not verbalize delirium, and had no cognitive impairment. She reported visual hallucinations. These hallucinations appeared several times a day for variable periods of time and lasted a few minutes. They caused anxiety and insomnia. The patient reported that she was embarrassed by these hallucinations. She saw unfamiliar human faces with no other associated signs. These images did not speak, and the patient knew that the hallucinations were unreal.

The neurologic exam revealed no abnormalities. Her Mini-Mental State Examination (MMSE) score was 28.

Ten days after transsphenoidal adenomectomy, the visual acuity of the patient was preserved and the intraocular pressure was normal. The slit lamp biomicroscopy showed no cataracts. The automatic visual field test revealed a bilateral visual field defect with temporal quadrantanopia.

Biological assessments were normal. The levels of all pituitary hormones were normal. The analysis in blood and urine did not detect the presence of toxic agents.

The brain magnetic resonance imaging showed no abnormalities apart from the expected postoperative changes of a cavity in the sella turcica.

In our case, the patient met all criteria of CBS. No other psychiatric disorder was suspected following the interview. Differential diagnoses such as neurologic disorders, psychiatric disorders, toxic causes, metabolic disorders, auditory and sleep deprivation, and hypnopompic or hypnagogic hallucinations were excluded by psychiatric and neurologic exams.

The patient was put on 5 mg of Olanzapine daily.

Hallucinations were gradually lowered and completely resolved after two weeks of treatment. The dose of 5 mg daily of Olanzapine was maintained for three months and then gradually reduced and discontinued over the next two months without the recurrence of the symptoms.

The last psychiatric follow-up was four months after the treatment withdrawal, and the patient was asymptomatic. She was satisfied with the care she received.

## 3. Discussion

CBS is a rarely reported clinical situation, but the real prevalence is still unknown for several reasons [3]. First, patients may not report hallucinations as they are scared to be considered to have a mental disorder.

Second, usually, patients do not consult a psychiatrist initially. In this case, inconsistent depth of questioning may lead to missing the diagnosis. Third, the diagnostic criteria of CBS have changed over time [1].

The pathophysiology of CBS remains debatable [4]. Several hypotheses had been advanced to explicate this phenomenon [4, 5]. The release theory and the sensory deprivation theory are the two most known mechanisms [4]. According to the first theory, visual hallucinations are due to a neural defect in the visual pathways [2]. False signals caused by the abnormal signal transmission may result in complex visual hallucinations when they are associated with normal visual activity [2]. Regarding the second theory, it was demonstrated that in healthy subjects, hyperexcitability in the visual cortex is blocked by the normal sensory input [6]. Thus, in visually impaired patients, sensory input reduction is incriminated in the appearance of hallucinations [6]. Up to the present, there is not enough argument to support either theory [4]. In the present case, the patient had a pituitary macroadenoma compressing the optic chiasma, which may support the first hypothesis.

Characteristics of visual hallucinations vary widely among patients with CBS, but they are commonly clear and well defined [7].

Different types of visual hallucinations have been described [1]. The underlying mechanism which defines the type of visual hallucination remains unclear [3]. The role of cultural and/or religious beliefs and education level are worth to be evaluated in this context.

Generally, patients with defects of the visual pathways, eye abnormality affecting the transmission of light, and diseases of the neuroretina may be concerned by this pathology [1]. To our knowledge, pituitary adenoma is rarely reported as an etiology of CBS [2, 8–10]. Park et al. [2] described a case of CBS that manifested in the first postoperative day after transsphenoidal adenomectomy for pituitary adenoma in a 46-year-old man.

It is important to point out that the neural mechanisms behind the onset of CBS in patients with pituitary adenoma are still unknown [3].

It is important to remember that CBS was also reported in patients with visual field defects and preserved central vision [11]. This situation concerns patients with glaucoma and was discussed by Subhi et al. [11]. In fact, central vision is affected only in patients suffering from severe glaucoma neurodegeneration [11]. The authors found that the prevalence of CBS in patients with glaucoma ranges between 2.8% and 20.1% and increases with the severity of visual impairment [11]. Although data concerning the prevalence of CBS in a representative sample of patients with glaucoma only are scarce, this pathology remains more frequently observed in patients with diseases that affect the central vision such as age-related macular degeneration [11, 12].

In conclusion, it is the first Tunisian case of CBS reported in English language. This peculiar condition seems to be under-recognized in our country.

Clinicians should be aware that visual hallucinations may concern patients without psychiatric disorders. In fact, it is important to distinguish CBS from other psychiatric diseases that may cause visual hallucinations since it has a different prognosis and it needs different managements.

## Data Availability

The data used to support the findings of this study are included within the article.
